# Mechanistic modeling of aberrant energy metabolism in human disease

**DOI:** 10.3389/fphys.2012.00404

**Published:** 2012-10-25

**Authors:** Vineet Sangar, James A. Eddy, Evangelos Simeonidis, Nathan D. Price

**Affiliations:** ^1^Institute for Systems BiologySeattle, WA, USA; ^2^University of Illinois, Urbana-ChampaignIL, USA; ^3^Luxembourg Centre for Systems Biomedicine, University of LuxembourgEsch-sur-Alzette, Luxembourg

**Keywords:** mitochondria, energy metabolism, constraint-based models, flux balance analysis, Warburg effect, systems biology

## Abstract

Dysfunction in energy metabolism—including in pathways localized to the mitochondria—has been implicated in the pathogenesis of a wide array of disorders, ranging from cancer to neurodegenerative diseases to type II diabetes. The inherent complexities of energy and mitochondrial metabolism present a significant obstacle in the effort to understand the role that these molecular processes play in the development of disease. To help unravel these complexities, systems biology methods have been applied to develop an array of computational metabolic models, ranging from mitochondria-specific processes to genome-scale cellular networks. These constraint-based (CB) models can efficiently simulate aspects of normal and aberrant metabolism in various genetic and environmental conditions. Development of these models leverages—and also provides a powerful means to integrate and interpret—information from a wide range of sources including genomics, proteomics, metabolomics, and enzyme kinetics. Here, we review a variety of mechanistic modeling studies that explore metabolic functions, deficiency disorders, and aberrant biochemical pathways in mitochondria and related regions in the cell.

## Introduction

Energy-producing metabolic pathways—including those primarily active in mitochondria—are among the most central components of eukaryotic life. As such, dysfunction in these pathways has been implicated in numerous human diseases, including diabetes, cancer, epilepsy, myopathy, cognitive impairment, and neurodegenerative disorders [reviewed in DiMauro and Schon (2003)]. Advances in high-throughput technologies have provided a wealth of measurements that, when effectively harnessed, can provide enormous insights into the underlying mechanisms that give rise to aberrant energy metabolism and associated diseases. Computational modeling, in particular, represents a powerful approach to interrogate metabolism on the systems level and to efficiently integrate heterogeneous data types such as genomic, transcriptomic, proteomic, and metabolomic. Here, we highlight some of the major efforts and successes thus far in modeling energy metabolism using an approach known as constraint-based (CB) modeling. We focus heavily on modeling studies of mitochondrial function, as this organelle houses many key metabolic processes related to energy production. Aside from models of mitochondrial metabolism and related pathways, we also review the Warburg effect, a long-established cancer phenomenon that has recently garnered significant attention.

Mitochondria are maternally-inherited, dynamic organelles present in all cells of the body, excluding the red blood cells. They vary in number from a few hundred to thousands per cell, depending upon the cell type and its functions. Mitochondria play a vital role in cell life, death, and differentiation. They are centers of adenosine triphosphate (ATP) production and a multitude of other housekeeping functions such as biosynthesis of amino acids, oxidation of fatty acids, and apoptosis. The mitochondrion has been appropriately called the “powerhouse” of the cell, and this function has been the focus of intensive research.

Each mitochondrion is a semi-autonomous organelle with its own DNA and replication machinery. The mitochondrial genome (mtDNA) is a 16,569 base pair long circular molecule (Anderson et al., 1981). Since its discovery, mtDNA has been sequenced across populations to investigate natural variation (Ingman and Gyllensten, 2006), and various mutations leading to disorders have been identified (Holt et al., 1988; Wallace et al., 1988; Vieira et al., 2000). The mtDNA encodes only 13 proteins, while the nuclear DNA (nDNA) encodes the rest of the mitochondrial proteins. The complete mitochondrial proteome was originally estimated to include 1500 proteins, based on quantitative two-dimensional gel analysis (Lopez et al., 2000). Later, Pagliarini et al. performed a comprehensive experiment utilizing mass spectrometry, GFP-tagging and machine learning to identify 1098 genes and respective mitochondria-localized proteins in 14 different mouse tissues (Pagliarini et al., 2008). This study revealed variation within each tissue in the *number* of mitochondria per cell as well as in mitochondrial gene and protein expression. For example, the cells in the heart have three times more mitochondria as compared to cells in the liver. It was further observed that while different tissues display variation in mitochondrial gene expression, developmentally related organs exhibit higher similarity in their mitochondrial transcriptome and proteome (Forner et al., 2006).

The availability of extensive genetic and proteomic information has made it possible to catalog the relationship between mutations in the mtDNA and nDNA and emergent disease phenotypes. Defective oxidative phosphorylation (OxPhos), for instance, has been implicated in various respiratory chain diseases, ranging from neonatal lethality to adult-onset of neurodegeneration (DiMauro and Hirano, 2006); Kirby and Thornburn cataloged mutations in 92 protein-encoding genes causing OxPhos-related diseases (Kirby and Thorburn, 2008). A similar study (Smits et al., 2010) noted that there are 200 documented mutations in the mtDNA and 100 in nDNA among OxPhos-related genes. In addition to defects in OxPhos genes, other mitochondrial genes have been implicated in diseases such as diabetes (DiMauro and Hirano, 2006), Miller syndrome (Ng et al., 2010), and neurodegenerative diseases [reviewed in Schon and Przedborski (2011)]. For example, mutations in the mtDNA replication protein, mtDNA polymerase γ, cause degeneration of the cerebellum (Hakonen et al., 2008).

The MitoPhenome database, which catalogs mutations in mtDNA with the resulting diseases, was generated by performing an extensive literature search (http://www.mitophenome.org). To date, the database includes 502 hierarchical clinical features with defects in 174 mitochondria-resident proteins. In addition to MitoPhenome, other mitochondrial databases such as MitoP2 (Elstner et al., 2008), MitoCarta (Pagliarini et al., 2008), MitoMiner (Cotter et al., 2004; Smith and Robinson, 2009), and MitoProteome (Cotter et al., 2004) have been curated, and these provide information regarding the mitochondrial genome and proteome in various organisms and tissues.

The abundance of available experimental data, particularly those coupling large-scale molecular measurements to resulting disease phenotypes, presents opportunities to achieve enhanced insight into mitochondrial physiology and disease. Computational modeling of metabolic functions in the mitochondria provides an advantageous means to combine different data types, and to investigate the effects of genomic and proteomic perturbations on the pathway and genome scales. In particular, constructing biochemically detailed models allows one to leverage existing literature and experimental knowledge—coupled with the enforcement of physical and chemical laws—to provide a functional context for high-throughput data. In this way, computational models can be used to help elucidate genotype-to-phenotype relationships in mitochondria and related diseases. CB modeling approaches applied to reaction networks in particular have been employed to achieve this goal. The basic premise of these approaches is that mathematical constraints are applied to a system, thereby defining an operating space that best represents the biological and environmental conditions of interest.

When enough information is available, one can construct a kinetic model, which establishes a highly constrained mathematical representation of the reaction network; kinetic modeling is a well-established approach to simulate mitochondrial function *in silico*. A kinetic model typically includes a set of ordinary differential equations or partial differential equations, describing the export and import of metabolites and the rate of change of metabolite concentrations, with associated parameters such as enzyme capacities and equilibrium or rate constants governing the metabolic processes. Kinetic models have been built to simulate various mitochondrial functions such as ATP production and beta-oxidation of fatty acids (Korzeniewski, 1998a,b; Cortassa et al., 2003; Beard, 2005; Modre-Osprian et al., 2009); the development of kinetic mitochondrial models to study metabolism and disease has recently been reviewed elsewhere (Cortassa and Aon, 2012). Although, kinetic modeling holds promise for its power to accurately capture biochemical dynamics, the unavailability of the necessary parameters such as rate constants, enzyme concentrations, initial and steady-state metabolite concentrations, and electrical potential impedes its implementation for many-component and highly connected biological processes.

Alternatively, stoichometric modeling is a more generalized, CB approach designed to simulate complex living systems by integrating data from multiple sources and quantitatively analyze the flux through a cascade of reactions to exhibit a specific phenotype. Given the widely employed use of “constraint-based (CB) modeling” in the systems biology community to denote stoichiometric modeling, we will henceforth consider this former term distinct from more detailed kinetic modeling. In contrast to kinetic modeling, CB modeling requires relatively few parameters and defines a set of non-excluded states that is consistent with the available data and applied physico-chemical constraints. As such, these models can be highly suitable to integrating and interrogating high-throughput datasets for a biological investigation of interest. Procedures for the construction and analysis of CB models are well established in microbial species (Feist et al., 2009; Thiele and Palsson, 2010), and these approaches have recently been gaining increased attention for the study of human metabolism and disease.

**Figure 1:**
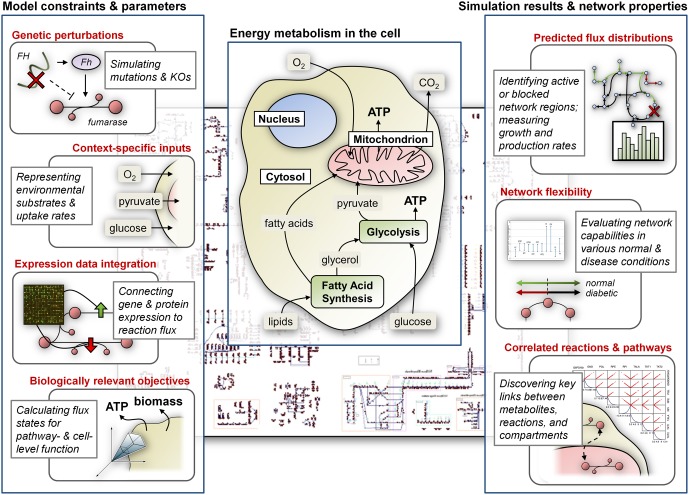
**CB modeling of energy metabolism.** Constraints and condition-specific parameters are most commonly represented as bounds on the flux through reactions in the network; optimizing for specific cellular objectives identifies network states that satisfy constraints and simulate *in vivo* or *in vitro* metabolism. Computational tools can be used to analyze models and investigate metabolic capabilities. The center panel highlights important components of cellular energy metabolism.

Here we present various applications of CB modeling to understand functioning of the mitochondria under normal and disease conditions (Figure 1). These studies include modeling of the tricarboxcylic acid (TCA) cycle, ATP production, and other processes in energy metabolism, with particular focus on how corresponding metabolic states relate to diseases such as diabetes, ischemia, α-ketoglutarate deficiency, and others (Ramakrishna et al., 2001; Vo et al., 2004; Thiele et al., 2005). While CB models of energy metabolism and associated diseases have predominantly focused on pathways of the mitochondria, recent studies have also more generally explored the implications of dysfunctional energy pathways in neurodegenerative diseases and in various cancers. In particular, we review models of Leigh's syndrome (LS) and Alzheimer's disease, as well as the Warburg effect in cancer. Importantly, some of the key processes that are simulated or highlighted may still be localized or related to the mitochondria, but these studies do not restrict their focus to the organelle and instead attempt to capture global cellular metabolism related to energy production.

## Overview of constraint-based modeling

CB modeling is a mathematical representation of the chemical reaction rates (i.e., flux) through metabolic reactions in a living system. The stoichiometry of the biochemical network is depicted in the form of a stoichiometric matrix ***S***, in which rows represent metabolites and columns represent reactions. For each reaction, the metabolites consumed (i.e., substrates) are represented by negative coefficients, whereas positive coefficients indicate production of metabolites; all metabolites that do not participate in a reaction are assigned a zero coefficient, resulting in a sparse matrix. ***S*** provides the relationship between reaction activity and metabolite concentration change as ***Sv*** = d***x***/dt, where the vector ***v*** represents the flux through each reaction, and concentrations of all metabolites are represented by vector ***x***. In this way, the numbers in ***S*** impose stoichiometric constraints on reaction fluxes, where the net change of each metabolite is a direct function of the reactions in which it participates. Often, the system is assumed to be in a net mass-balanced quasi-steady state that is represented by ***Sv*** = 0. Lower and upper bounds (*v*_min_ ≤ ***v*** ≤ *v*_max_) are implemented to enforce directionality and capacity constraints for each reaction.

In mathematical terms, the constraints define a multi-dimensional solution space of flux distributions for a specified condition that are consistent with the available data. While additional constraints such as metabolite concentrations and localization can further shrink the solution space, the system typically remains under-determined, with many alternative flux distribution solutions that satisfy ***Sv*** = **0**. By optimizing a relevant objective function, thereby driving network flux toward the production or consumption of a defined set of metabolites, a single distribution ***v*** that satisfies the objective can be found. Often, an objective function is chosen to replicate the assumed *in vivo* biological selection pressure, whether this is maximum ATP production, maximum biomass production or other. In practice, the objective function is specified by vector ***c***, with weights representing the relative contribution of each reaction. Linear programming can then be used to identify an optimal flux distribution that satisfies all constraints. Notably, there are typically many equivalent optimal solutions, and so the internal state of the network found to be optimal is usually not unique (Papin et al., 2002; Price et al., 2002; Reed and Palsson, 2004). The exercise of constraining the flux through a metabolic network using the ***S*** matrix, steady-state assumption, and defined bounds, along with calculating the optimal distribution of flux through all reactions for a given objective function, is called Flux Balance Analysis (FBA). This approach can be summarized mathematically as follows:

Maximize cTvsubject to:   Sv = 0   vmin ≤ v ≤ vmax

One of the major advantages of FBA is that it can be applied to large-scale metabolic networks, where a phenotype is an end result of activities of many biochemical reactions. A flux through each reaction can be calculated, and patterns of consumption and production of each metabolite can be predicted, even for systems with tens of thousands of components. Additional methods such as Flux Variability Analysis (FVA) (Orth et al., 2010) or Monte Carlo sampling of solution spaces (Almaas et al., 2004; Price et al., 2004) can address the variability possible in each of these computed reaction fluxes as well. Importantly, kinetic information such as enzyme concentrations is not required, although this information—when incorporated into more advanced FBA-based frameworks—will certainly facilitate accurate calculation of the *in vivo* relevant flux distributions. Another advantage is that FBA can be used to perform simulations under varied conditions simply by altering the constraints such as substrate availability or ATP production.

The accuracy of CB models depends in part on how well the ***S*** matrix represents the actual biochemical network of the living system. Therefore, construction of ***S*** for a specific organism is an important, data-intensive, and often arduous procedure. The process starts with collecting species-specific data from various sources such as genome annotations, high-throughput experiments, and relevant literature of the biochemistry and physiology of the organism. This leads to a draft network that must then be gap-filled (Reed et al., 2006; Kumar et al., 2007) and updated until it can be used to simulate phenotypes, thereby establishing a computable *in silico* model. For example, predicted flux through a defined biomass reaction can be interpreted as cellular growth; likewise, environmental or genetic conditions that block flux through this reaction are considered “lethal.” With a computable model, predictions of phenotypes (based on reaction fluxes) such as knockout lethality, growth rates on different substrates, and/or byproduct section rates can be compared to experiment, and the model can thus be iteratively tested and improved. Context-specific information is then imposed on the network to either simulate different conditions *in silico*, or—for higher organisms—to establish a representative model for some subset of the global species network (Becker and Palsson, 2008; Jerby et al., 2010; Rolfsson et al., 2011). For example, for a mitochondrial model, only those reactions and metabolites localized to or associated with mitochondria are utilized; for a brain-specific model, only metabolic enzymes corresponding to genes expressed in brain tissue are used. The context-defining constraints are imposed as upper and lower bounds on reactions in the model, or by the inclusion or removal of specific genes and reactions. These bounds and the determination of whether network components are present or absent can be based on experimentally calculated values, or inferred through a computational approach, such as those available in the COBRA Toolbox (Orth et al., 2010). The main objective of all constraints applied to the reaction network is to simulate *in vivo* conditions as precisely as possible. With the development of better high-throughput measurement tools, and the consequent generation of more plentiful and more precise data, the ***S*** matrix and associated constraints will become more accurate.

## Modeling mitochondrial metabolism and disease-related aberrations

In this section, we review studies that focus on CB models of normal and malfunctioning mitochondria. As described above, the exercise typically starts with building a framework of reactions and applying constraints to mirror normal or disease states. After a model has been sufficiently refined, an objective function is established (e.g., ATP production), and FBA is performed to simulate mitochondrial function.

In 2002, Ramakrishna and Palsson characterized the optimal flux distributions for maximal ATP production in a mitochondria-specific metabolic network, which included glycolytic pathways, the citric or TCA cycle, and the electron transport system (ETS). ATP production and the concomitant O_2_ uptake rates were then simulated for three common substrates: glucose, lactate, and palmitic acid. Their results showed that the model could uptake glucose to produce 38 moles of ATP with the concomitant utilization of 6 moles of O_2_; lactate uptake corresponded to 17.5 moles of ATP with the utilization of 3.0 moles of O_2_, and palmitic acid uptake (a 14-C fatty acid) led to 129 moles of ATP, but required 23 moles of O_2_, which is in agreement with experimental values.

Extending the analysis further, the authors simulated changes in ATP production as a result of mutations in genes encoding TCA cycle enzymes (i.e., by constraining flux through associated reactions to zero). Mutations in TCA enzymes such as α-ketoglutarate dehydrogenase, fumarase, and succinate dehydrogenase have been implicated in mitochondrial diseases (Bourgeron et al., 1994; Rustin et al., 1997). The authors also simulated a loss of activity for each enzyme individually by sequentially inactivating the corresponding reaction in the TCA cycle. Deletions of all sequential enzymes led to no production of ATP. However, with only the α-ketoglutarate dehydrogenase deletion, the model predicted a reduced ATP yield and excess production of α-ketoglutarate. The results of this simulation were in agreement with experimental observations (Rustin et al., 1997).

The study presented by Ramakrishna and Palsson focused entirely on mitochondrial processes involved specifically in ATP production. In 2004, Vo et al. constructed an enhanced metabolic model, in which they expanded to additional mitochondrial functions. In addition to ATP production, they also studied heme and mixed phospholipid biosynthesis, utilizing proteomics, and biochemical data from human cardiac mitochondria (Ozawa et al., 2003; Taylor et al., 2003).

The resulting model included 189 reactions and 230 metabolites, and all reactions were elementally and charge balanced. The list of processes included the TCA cycle, OxPhos, fatty acid β-oxidation, phospholipid biosynthesis, urea cycle, and reactive oxygen species (ROS) detoxification. Each reaction and metabolite was assigned to its respective compartment such as mitochondria, extracellular space, or cytosol. In addition to the localization data, a confidence score was assigned to each reaction based on the data supporting it. For example, the highest confidence of 4 was assigned to a reaction supported by biochemical data, and the lowest confidence (0) to reactions with *in silico* evidence only. One of the salient features of the model was that the majority of the reactions in the network were assigned high confidence scores, indicating that the model was experimentally well supported.

This model was used to simulate ATP production and calculate yield from three energy sources: glucose, fatty acids, and amino acids. The model produced 31.5 ATPs for each mole of glucose. For calculating ATP yield from fatty acids, the authors focused only on palmitate and calculated that each mole of palmitate led to production of 106 ATPs, which is different from the value of 129 reported in the literature (Lehninger et al., 1993). While calculating ATP yield from amino acids, glutamate was selected as the substrate; the yield was calculated to be 20.5 ATPs/mol of glutamate. Results for heme biosynthesis, amino acid metabolism and phospholipid simulations were similar to the experimental observations (Sedman et al., 1982) further validating the model.

In addition to modeling biological processes, the authors studied network characteristics such as network flexibility and correlated reaction sets. In order to analyze flexibility, FVA was performed. FVA calculates minimal and maximal boundaries for fluxes through each reaction in the network, such that the optimal solution is still satisfied. This analysis revealed that the solution space for optimal ATP production is restricted compared to heme and phospholipid biosynthesis objectives, indicating that the former is a highly coordinated biological function with a narrower set of possible network states satisfying optimal production. Another interesting aspect that was investigated in this study was the exploration of correlated sets of reactions. While calculating alternate optima, authors identified a set of reactions with highly correlated flux distributions across various objective functions representing multiple mitochondrial functions. These highly correlated sets are candidates to be potentially co-regulated in the cell.

A subsequent study (Thiele et al., 2005) further refined this mitochondrial metabolic model and used it as a tool to assess metabolic states by integrating available experimental measurements under different physiological conditions: normal, diabetic, ischemic, and different diets. They also studied the impact of suggested treatments on mitochondrial energy metabolism under diabetic conditions. The authors employed linear programming and uniform sampling techniques to identify candidate steady states and to characterize network properties under physico-chemical and experimentally defined constraints.

One of the key modifications made in (Thiele et al., 2005) to the previous mitochondrial model was the addition of ketone body degradation reactions. This addition was important because diabetes leads to high concentrations of ketone bodies in the blood. In simulations, this study generated candidate steady-state flux distributions for different metabolic states, such as diabetes, ischemia, normal, and different diets. Different states were classified based on the experimental or measured values of reactions exchanging (uptake or secretion) metabolites between the cell and the surrounding environment; these values determined constraints on the exchange reactions in the model, thereby enforcing the different metabolic states. For example, for simulating diabetes, mitochondrial fatty acid uptake through the carnitine palmitoyl-transferase (CPT-1) shuttle was increased, which reflects the increased uptake observed in diabetes. Similar constraints, such as normalized glucose or normalized ketone body uptake rates, were applied to reflect other metabolic states. This was an excellent example of how CB modeling can be used to simulate intracellular conditions in normal and diseased states.

Using uniform sampling of the steady-state flux space for the four constrained mitochondrial models, the authors calculated the flux distribution (in this case, the frequency of observed flux values over all random samplings, as opposed to the set of fluxes calculated in one instance of FBA for all reactions in the network) for various reactions such as lactate exchange, ATP demand, oxygen exchange, and others. The sampling technique produced a range of allowable metabolic network states that represented possible steady-state flux distributions of the network for all four states, defined using constraints from experimentally measured conditions. Results of this analysis showed that there is reduced network flexibility in diabetes, and changes in oxygen levels or ATP can greatly affect mitochondrial metabolism.

One of the unique features of this study was that the authors modeled therapeutic approaches for ischemia and predicted the effectiveness of these approaches based on network simulations. Predictions agreed remarkably well with the observed side effects of these therapies; for example, one of the therapeutic approaches suggested previously for ischemia was to increase glucose uptake (Apstein, 1998) in the hopes of increasing the availability of ATP. However, the analysis of candidate flux distributions predicted that this therapy would not change the disease metabolic state, but rather would lead to higher production of lactate, which was the known side effect.

Additionally, the model predicted that the oxygen requirements for the cardiac myocyte were much more constricted to be near maximal under typical diabetic conditions. This finding provided a possible mechanistic explanation for the clinical observation that ischemic events, which limit oxygen, are more likely to be fatal for patients with diabetes than for unaffected people (Taegtmeyer et al., 2002). Another intriguing outcome of this analysis was that flux through pyruvate dehydrogenase was predicted to be near zero under typical diabetic conditions. The gene encoding pyruvate dehydrogenase had earlier been found to be a highly differentially expressed gene in transcriptomics studies (Stanley et al., 1997). From transcriptomics analyses alone, it can be difficult tell the difference between gene expression changes that are causal or passenger—or, in particular, what changes might lead to a restoration of the system. In this example, the model clearly suggested that the much lower expression value of the pyruvate dehydrogenase-encoding gene is a down-regulation response of the cell, which is appropriate to the metabolic conditions in the surrounding blood of a typical diabetes patient. Thus, engineering, for example, an increase in the expression of this gene to restore it to its normal state would be doomed to fail—there are actually no possible steady-state flux distributions in the mitochondrial metabolic network for which this reaction could be used, given the availability of surrounding metabolites. This is one example of how mechanistic models can help decipher what might be causal disease perturbations, and what might be potential therapeutic interventions, from those that are not. They can also help to discern what changes in the expression state of a system might be a disease *perturbation* from what seems to be an appropriate *response* of the system to compensate for the perturbation.

More recently, a heart mitochondrion model (*i*AS523) was reconstructed (Smith and Robinson, 2011), in which metabolites were made part of the model only if they were consumed or produced in the mitochondria. Information from MitoMiner, a mitochondrial proteomics database, was utilized for establishing the address of each metabolite in the cell. Constraints applied to the model were chosen from the literature to mimic conditions specific to the heart. The model was then refined iteratively by incorporating information from multiple sources. For testing the predictive abilities of the model, the authors simulated three disorders of, or related to, the TCA cycle: (1) fumarase deficiency (OMIM: 606812), (2) succinate dehydrogenase deficiency (OMIM: 252011), and (3) α-ketoglutarate dehydrogenase deficiency (OMIM: 203740). The authors ran simulations for various mitochondrial functions with constraints specific to each disorder. The model predicted ATP production accurately for all diseases, especially for the fumarase deficiency disorder.

**Figure 2:**
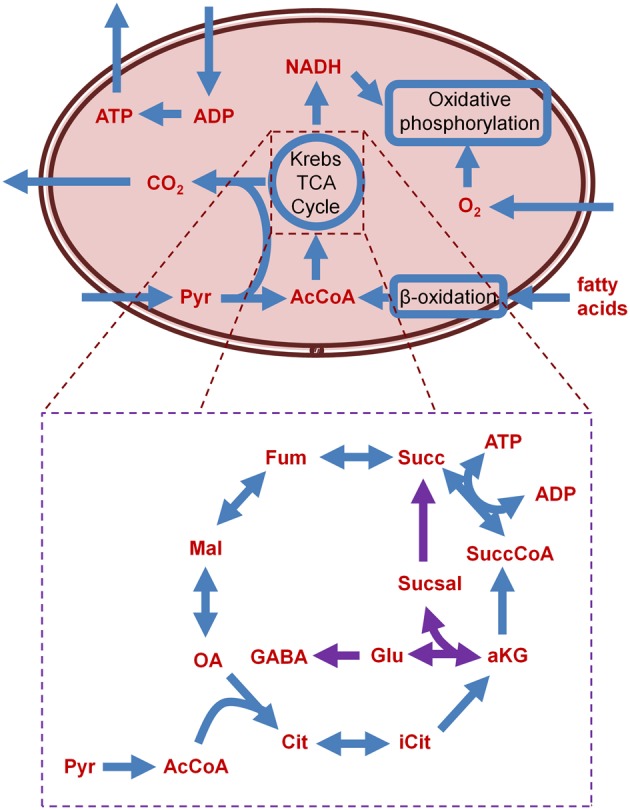
**Schematic of the major metabolic processes housed in the mitochondria, and detail of the TCA cycle and the GABA shunt (GABA shunt related reactions in purple).** AcCoA, acetyl coenzyme A; ADP, adenosine diphosphate; aKG, α-ketoglutarate; ATP, adenosine triphosphate; Cit, citrate; Fum, fumarate; GABA, γ-aminobutyric acid; Glu, glutamate; iCit, isocitrate; Mal, malate; NADH, nicotamide adenine dinucleotide; OA, oxaloacetate; Pyr, pyruvate; Succ, succinate; SuccCoA, succinyl-coenzyme A; Sucsal, succinate semialdehyde.

In fumarase deficiency disorder, fumarase (fumarate hydratase) is completely shut down; the TCA cycle thus ends at the step where fumarate would normally be converted to malate by fumarase, leading to accumulation of the former. The TCA network structure with this disorder precludes ATP production in the heart, however, patients with this disorder do not present with heart defects. To investigate this discrepancy, authors simulated elevated metabolism by removing the boundary constraints on all metabolites. Through this process, they identified that increased concentration of certain metabolites, such as glutamate, aspartate, or valine, lead to increased ATP production in the TCA cycle. This result agrees with findings in the literature (Bourgeron et al., 1994), suggesting that there is a bypass through which metabolites are exported out of the mitochondrial matrix into the cytosol. The malate-aspartate shuttle (Figure 2) is the bypass reaction that exports metabolites out of the mitochondria. Because of this bypass, fumarase deficiency does not fully shut down the TCA cycle, but instead only reduces ATP production. The effect of aspartate uptake was even more pronounced than the increased uptake of glucose, suggesting that providing excess of the former may best reduce the effect of fumarase deficiency. Increasing the uptake of asparate and serine was suggested as a therapeutic strategy, but it remains to be seen if the aspartate uptake levels can be replicated *in vivo*; however, this *in silico* result is an experimentally testable hypothesis.

## Modeling energy metabolism in neurodegenerative disorders

Defects in energy metabolism have been implicated in various neurodegenerative diseases such as LS, Alzheimer's disease, and Parkinson's disease. Here, we present modeling efforts studying neurodegenerative diseases in relation to defects in ATP production in mitochondria.

In 2007, Vo et al. published a model for LS and suggested a framework for employing CB modeling to recommend a treatment strategy for this disease. The authors modeled the status of mitochondrial processes in LS by constructing a model for LS-affected fibroblasts and normal fibroblasts, utilizing experimental measurements such as substrate uptake, metabolite secretion rates, and isotopomer data. The authors experimentally grew normal and LS cells in media with ^13^C-labeled glucose and identified the distribution of metabolites containing labeled glucose through gas chromatography-mass spectrometry (GC-MS). The study revealed that although LS-affected cells have slower metabolism as compared to normal cells, their ATP production rate was similar to that of the normal cells. Additionally, LS-affected cells exhibited a narrower flux range for the reactions involved in ATP production. This work implicated mutations in succinate cytochrome c reductase and defective respiratory complex II as the most likely candidates for inducing LS in the cell line, ruling out malfunction of ATP synthase, pyruvate dehydrogenase complex, or respiratory complexes I, III, and IV as likely causes of the disease. Another impressive aspect of this study was that the authors suggested an effective vitamin treatment for LS patients. Predictions of the model were confirmed *in vitro* with experimental measurements in both cell lines. This study is a prime example of how the inclusion of a multitude of experimental data can enhance the accuracy of *in silico* models and can suggest an effective treatment regime for a complicated disease such as LS.

Mitochondria-centric network models have been reconstructed now for three different subtypes of neurons (Lewis et al., 2010), and used to simulate the relationship between Alzheimer's disease and the TCA cycle. Utilizing genomics, proteomics and literature-based data, Lewis et al. curated three cell type-specific canonical models representing cholinergic, GABAergic, and glutamergic neurons that were coupled to astrocytes via interstitial space; neuron and astrocyte metabolism were subdivided into cytosolic and mitochondrial compartments. This was the first large-scale CB modeling of multicellular metabolic interactions between astrocytes and neurons, and also the most detailed model for brain energy metabolism to date. Authors simulated production of ATP to test the accuracy of the model, and calculated the predicted values to be within 8% of published values. To investigate the previously reported relationship between Alzheimer's and distorted activity of TCA cycle enzymes (Reiman et al., 2004), the authors modeled the effect of mitochondrial α-ketoglutarate dehydrogenase (AKGDm) mutation on ATP production in all three models.

Results showed impaired OxPhos in cholinergic and glutamergic neurons; however, the GABAergic neuron model did not show any decrease resulting from the simulated AKGDm dysfunction. Investigating further, the model predicted that the GABA shunt (Figure 2) was carrying the flux obstructed by mutated AKGDm. The GABA shunt includes a gene glutamate decarboxylase (GAD), and analysis suggested that isoforms of GAD could provide protection to GABAergic neurons. In these neurons, GAD carried flux, whereas in the other two neurons, GAD was absent, entailing greater susceptibility to dysfunctional AKGDm. These results were corroborated with expression data from Alzheimer's-affected brain regions, where underexpression of GAD was found in the affected regions, and overexpression in the unaffected regions. Pathway analysis on the microarray data from different regions of the Alzheimer's-affected brain revealed that four regions (posterior cingulate cortex, middle temporal gyrus, hippocampus, and entorhinal cortex)_had lower metabolic rates in Alzheimer's disease also showed decreased rates of glycolysis and TCA.

In addition to TCA modeling, Lewis et al. investigated the relationship between acetylcholine and mitochondrial metabolism. Studies have shown that the cytosolic acetyl-CoA needed to produce the neurotransmitter acetylcholine originates in the mitochondria. Earlier, Watson and Craft (2004) had reported that an increase in glucose uptake leads to improved cognition in Alzheimer's patients, further demonstrating a relationship between energy metabolism in the mitochondria and the generation of cytosolic acetylcholine. Using CB modeling, authors identified two pathways that were involved in the indirect transport of mitochondrial acetyl-CoA into the cytosol. Specifically, singular value decomposition was applied to a collection of 21,000 feasible reaction sets, yielding three pathways that allow for coupling of mitochondrial acetyl-CoA and cytosolic acetylcholine; two of these pathways, involving citrate or acetoacetate as carriers, respectively, were supported by omics data and enzyme localization. Through random sampling, the CB model showed a strong correlation between mitochondrial pyruvate dehydrogenase and cytosolic choline acetyltransferase, the enzyme that produces acetylcholine from acetyl-CoA, consistent with the experimentally observed coupling (Ragozzino et al., 1998). Furthermore, reaction fluxes in each of the two acetyl-CoA transport pathways were also significantly correlated with choline acetyltransferase. Using their model, Lewis et al. were able to identify potential connecting pathways between mitochondrial metabolism and acetylcholine production, and subsequent analysis also revealed that cholinergic neurotransmission likely depends on redundancy among these pathways.

Taken together, this study presented a workflow of constructing a model through an iterative process of hypothesis generation and incorporation of experimental data, leading to neuron models coupled to astrocytes with superior predictive power.

## Modeling the warburg effect in cancer

The Warburg effect, named after Otto Warburg, has been observed in many cancers and fast dividing cells, and is characterized by a switch from OxPhos—the normal route of energy production—to a combination of OxPhos and aerobic glycolysis. Glycolysis is a relatively inefficient way of producing ATP, typically utilized in the absence of oxygen, and leads to production of lactic acid. Although the Warburg effect was reported in 1956 (Warburg, 1956), the biological reasons and advantages of this switch to increased glycolysis in cancerous cells, even when oxygen is available, remain unclear. It has been proposed that aerobic glycolysis leads to increased biomass production, which would facilitate rapid proliferation of cancerous cells [reviewed by Vander Heiden et al. (2009)]. Lately, CB modeling of global metabolic processes—including both mitochondrial and cytosolic pathways—has been employed by various researchers to help reveal the underlying mechanisms for the Warburg effect in proliferating cells.

In 2010, a model simulating the Warburg effect (Vazquez et al., 2010) showed that aerobic glycolysis is favored by rapidly dividing cells because of (1) high glucose uptake capacity and (2) the solvent capacity of components allocated by the cell to OxPhos. Specifically, the authors explained the solvent capacity constraint as the maximum volume of the cell that is available for OxPhos enzymes. Higher glucose uptake will force the allocation of more resources toward ATP production; however, because of the solvent capacity constraint, the cell cannot increase the concentration of enzymes in the mitochondria, so this leads to inefficient production of ATP via glycolysis reactions in the cytosol. The author explained the solvent capacity constraint as the maximum volume of the cell that is available for OxPhos enzymes. This suggested that limited volume forces the cell to switch to aerobic glycolysis. Vazquez and Otalvi later argued that the cell instead switches to aerobic glycolysis because, for rapidly dividing cells, ATP demand is high and aerobic glycolysis provides higher ATP yield per volume density (Vazquez and Oltvai, 2011). They posited that energy demand is the only driving force for the existence of the Warburg effect in rapidly proliferating cells and argued against the need for metabolite precursors or biomass for the switch to aerobic glycolysis. Both studies were focused mainly on catabolic efficiency as the driving force behind the Warburg effect, and the Vazquez model was limited to energy metabolism reactions.

In 2011, Shlomi et al. used a genome-scale model of human metabolism (Duarte et al., 2007) to focus both on anabolic and catabolic aspects of the Warburg effect (Shlomi et al., 2011). Their model predicted three distinct growth states as suggested by the experimental observations (Ramanathan et al., 2005): (1) low OxPhos with almost no glycolysis; (2) high OxPhos with low glycolysis; and (3) low OxPhos with high glycolysis. The first and third states had already been reported; however, the notable feature of these predictions was the second state, which was a hybrid of normal cell energy metabolism and aerobic glycolysis. Utilizing a previously constructed model of *global* human metabolism (Duarte et al., 2007) to focus on this mitochondrial process, authors constrained the model by using enzyme concentrations that were computed from catalytic rates reported in published literature. Using different glucose uptake rates, energy production was maximized, and the results showed that it is cost-effective for the cell to switch to aerobic glycolysis when glucose uptake is unlimited. Cost was calculated as the enzyme concentration required per unit of glucose uptake. Authors computed the cost efficiency by comparing the ratio of ATP yield to cost under high glucose and optimal glucose uptake. Quantitatively, even though there was high growth (optimum glucose uptake condition), the cost to produce that growth was also high; for low growth (unlimited glucose uptake condition), the cost associated to produce growth was also very low. This was an interesting observation suggesting that the Warburg effect is an adaptation by a cell to be more efficient in utilizing the available resources both in catabolism and anabolism. This study also showed that by adding more biochemical constraints to the model, the accuracy of CB modeling could be increased, and the inherent strength of CB modeling in simulating a living system through linear equations could be exploited.

## Conclusions

Metabolic processes involved in energy production, especially those localized to mitochondria, have been extensively studied in relation to multiple pathologies. CB modeling has been applied to help understand the intricacies of biological processes in normal and deviant mitochondria, and provides a promising approach with potential to ultimately help guide therapeutic approaches for diseases. In the future, with the increased availability of more detailed experimental data, such as proteomic and metabolomics data, models will be refined, and their predictive power will be enhanced significantly.

**Figure 3:**
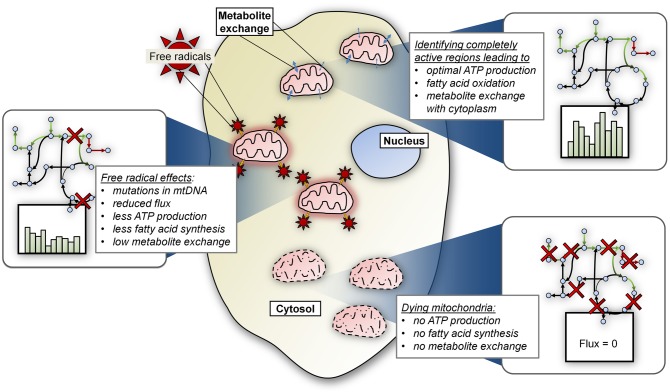
**Future areas of focus for CB modeling of mitochondrial metabolism.** Depicted here are several potential research areas that will be explored in future modeling endeavors, including the effects of (1) metabolite exchange between mitochondria and other compartments; (2) dynamic mitochondrial populations, in terms of size and age; and (3) free radical induced mutations in mitochondrial DNA.

Additionally, we expect that several other research areas (Figure 3) will enhance the connections between mitochondrial models and human disease. For example, models simulating the effects of the free radicals on mitochondrial functions, especially energy production, will likely mature into a valuable avenue of research. Free radicals cause mutations in mitochondrial DNA and mitochondria accumulate mutations as they age. Data-driven and mechanistic models that can simulate the effects of free radicals on mitochondrial functioning should further illuminate the pathogenic effect of resulting mutations on the emergence of disease. We also expect that CB models will be developed to more rigorously model the effects of inter-organelle metabolite trafficking on energy metabolism in mitochondria. Metabolite trafficking between mitochondria and other organelles, such as the endoplasmic reticulum, microsomes, and others, plays a critical role in energy metabolism in the cell. Normal functioning of a cell is contingent upon the dynamic exchange of metabolites between these organelles, and errors in transport can cause disease (Papin et al., 2002; Price et al., 2002).

Until now, models have considered mitochondrial processes in terms of representative activity of a single mitochondrion. In the future, we expect CB modeling to be used to simulate changes in the size and characteristics (e.g., age of mitochondria) of heterogeneous mitochondrial populations in the cell, in order to analyze effects on energy production from dynamic changes in mitochondria in cells. Changes in mitochondrial population, as well as errors in their turnover in the cell, have been implicated in several diseases (Schon and Przedborski, 2011). Finally, as the CB modeling field evolves, strategies will develop to simulate behavior of entire cell populations in organs or organ systems in disease and normal states, as well as in response to therapeutics. This will include simulating mitochondrial and other energy-related functions in different cell types in the same organ, and could incorporate information regarding various morphological features such as mitochondrial and/or cell density, extracellular matrix composition, and physico-chemical characteristics such as availability of metabolites.

Focus areas mentioned above either singularly or cumulatively have the potential to reveal the intricacies of energy metabolism in mitochondria and in relation to emergent physiology and disease. The goal of CB modeling is to accurately and comprehensively simulate facets of energy metabolism and its effect on disease. Models with high predictive accuracy require accurate and precise context-specific biological measurements. As technology and experimental procedures continue to improve, providing more data and at higher resolution, CB modeling will present an important avenue to contribute toward the understanding and the treatment of energy and mitochondrial metabolism-based diseases.

### Conflict of interest statement

The authors declare that the research was conducted in the absence of any commercial or financial relationships that could be construed as a potential conflict of interest.
